# Supplementary material for: A Volumetric Absorptive Microsampling UPLC-MS/MS Method for Simultaneous Quantification of Tacrolimus, Mycophenolic Acid and Creatinine in Whole Blood of Renal Transplant Recipients
Source: Pharmaceutics. 2022 Nov 22;14(12):2547. doi: 10.3390/pharmaceutics14122547 (PMC9788011; doi:10.3390/pharmaceutics14122547)
Supplement: Supplementary file 1 [file pharmaceutics-14-02547-s001.zip › pharmaceutics-1950652-supplementary.pdf]

Table S1. Basic information of transplant recipients and analysis results.

Details	Result
Number of samples	40
Male to Female	24/16
Age (year)	33.50 (27.50-38.00)
Transplant time (days)	199.00 (60.00-394.50)
TAC measured in WB (ng/mL)	5.80 (4.97-7.25)
TAC measured in VAMS (ng/mL)	5.15 (4.47-6.49)
TAC measured in DBS (ng/mL)	5.17 (4.32-6.19)
MPA measured in PL (μg/mL)	3.23 (2.52-4.15)
MPA measured in VAMS (μg/mL)	1.83 (1.27-2.31)
MPA measured in DBS (μg/mL)	1.65 (1.25-2.27)
Cre measured in SE (nmol/mL)	115.50 ± 33.03
Cre measured in VAMS (nmol/mL)	61.14 ± 22.93
Cre measured in DBS (nmol/mL)	65.47 (49.75-83.56)

Table S2. Data analysis and results during clinical validation of three analytes

Analyte		95%CI intercept by Passing-Bablok regression	95%CI slope by Passing-Bablok regression	Predictive performance	Intraclass correlation coefficients(ICC)	Mean % bias (95% CI) with Bland-Altman	Percentage of samples within acceptable limits of APE	Percentage of samples within clinically acceptable limits
TAC	VAMS vs WB	0.70(-0.05 - 1.31)	0.97(0.86 - 1.11)	RMSE:0.63 ng/mL<br>MPPE:0.12%<br>MAPE:5.87%	0.93	1.80(-1.50, 5.16)	95.00%	90.00%
	DBS vs WB	0.19(-0.61 - 0.78)	1.07(0.95 - 1.23)	RMSE:0.90 ng/mL<br>MPPE:0.03%<br>MAPE:4.90%	0.88	6.20(-2.45, 8.27)	82.50%	77.50%
MPA	VAMS vs PL	0.04(-0.33 - 0.35)	1.87(1.70 - 2.11)	MPPE:0.00%<br>MAPE:9.13%		-		
	corrected VAMS vs PL	0.10(-0.27 - 0.35)	0.98(0.88 - 1.10)	RMSE:0.44 μg/mL<br>MPPE:0.00%<br>MAPE:8.58%	0.97	-1.50(-6.37, 3.47)	88.57%	71.43%
	DBS vs PL	0.34(-0.01 - 0.69)	1.69(1.42 - 1.97)	MPPE:-1.44%%<br>MAPE:10.10%		-		
	corrected DBS vs PL	0.11(-0.29 - 0.46)	0.96(0.82 - 1.12)	RMSE:19.14 nmol/mL<br>MPPE:-1.13%<br>MAPE:9.22%	0.93	1.30(-5.17, 7.81)	71.43%	62.86%

Cre	VAMS vs SE	24.25(10.72 - 36.47)	1.46(1.26 - 1.68)	MPPE:0.02%<br>MAPE:7.36%		-		
	corrected VAMS vs SE	1.59(-14.87 - 17.66)	0.99(0.84 - 1.12)	RMSE:13.01 nmol/mL<br>MPPE:0.00%<br>MAPE:7.89%	0.92	-1.50(-4.87, 1.92)	92.50%	85.00%
	DBS vs SE	32.19(9.32 - 43.71)	1.21(1.01 - 1.58)	MPPE:-1.44%%<br>MAPE:10.10%		-		
	corrected DBS vs SE	1.44(-30.66-17.27)	0.99(0.83 - 1.29)	RMSE:19.14 nmol/mL<br>MPPE:-1.13%<br>MAPE:9.22%	0.84	2.90(-2.45, 8.27)	80.00%	70.00%

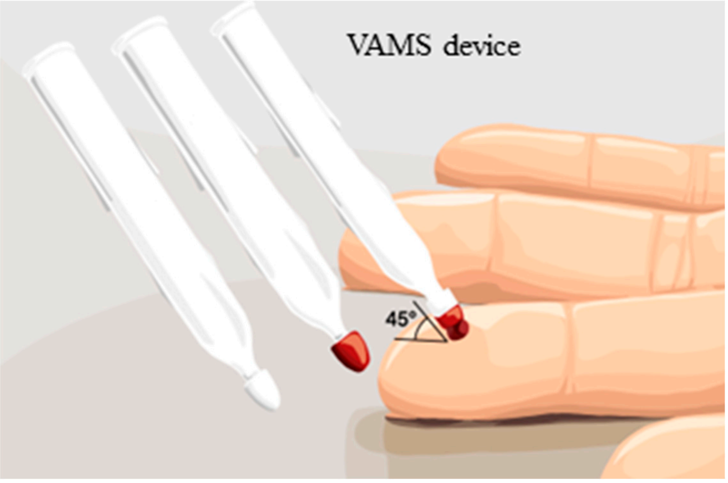

Figure S1. The working diagram of volumetric absorptive microsampling.

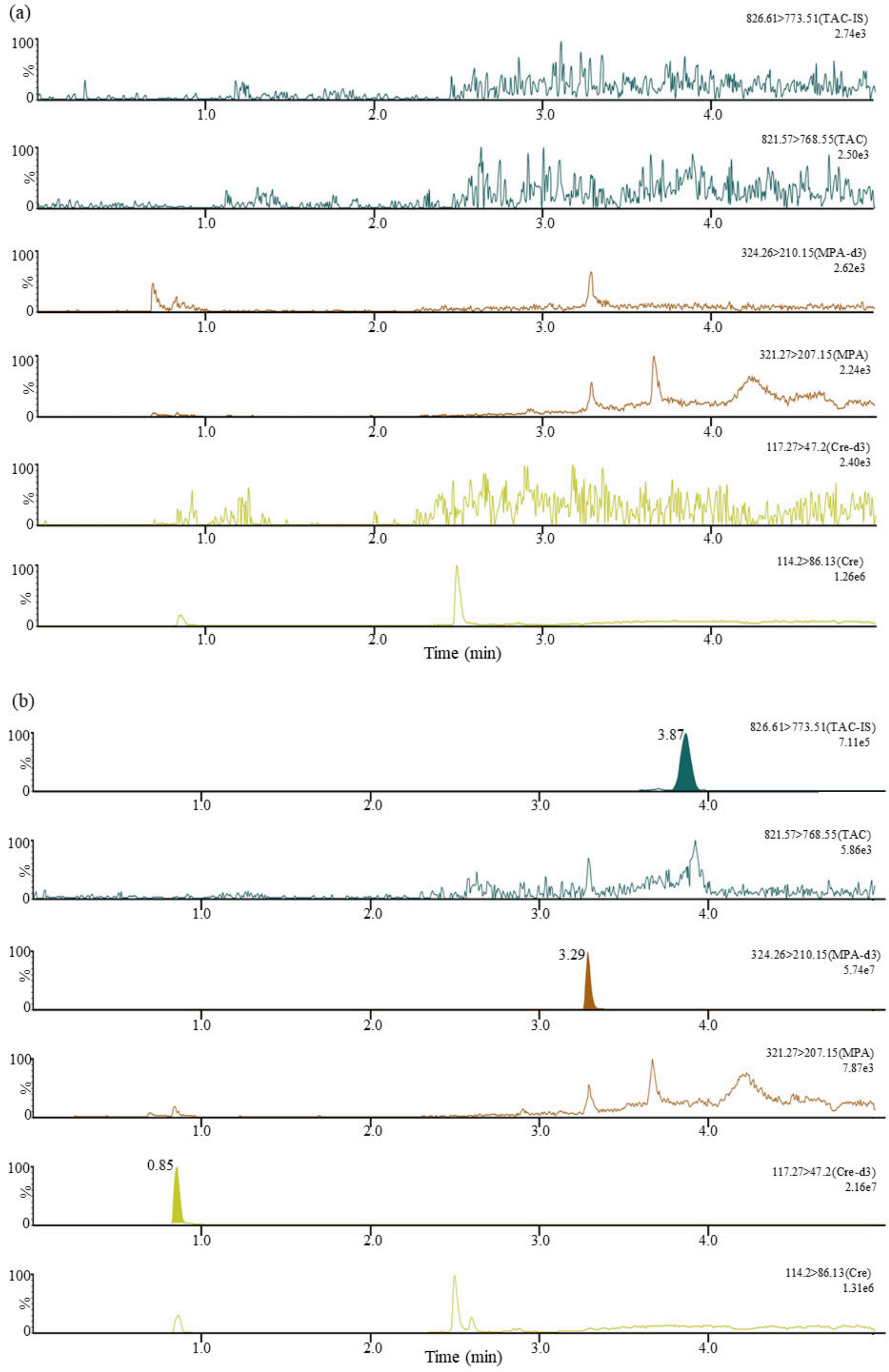

Figure S2. Representative ion chromatogram peaks. (a) the blank sample (with endogenous creatinine); (b) the blank sample spiked with the internal standard (with endogenous creatinine).

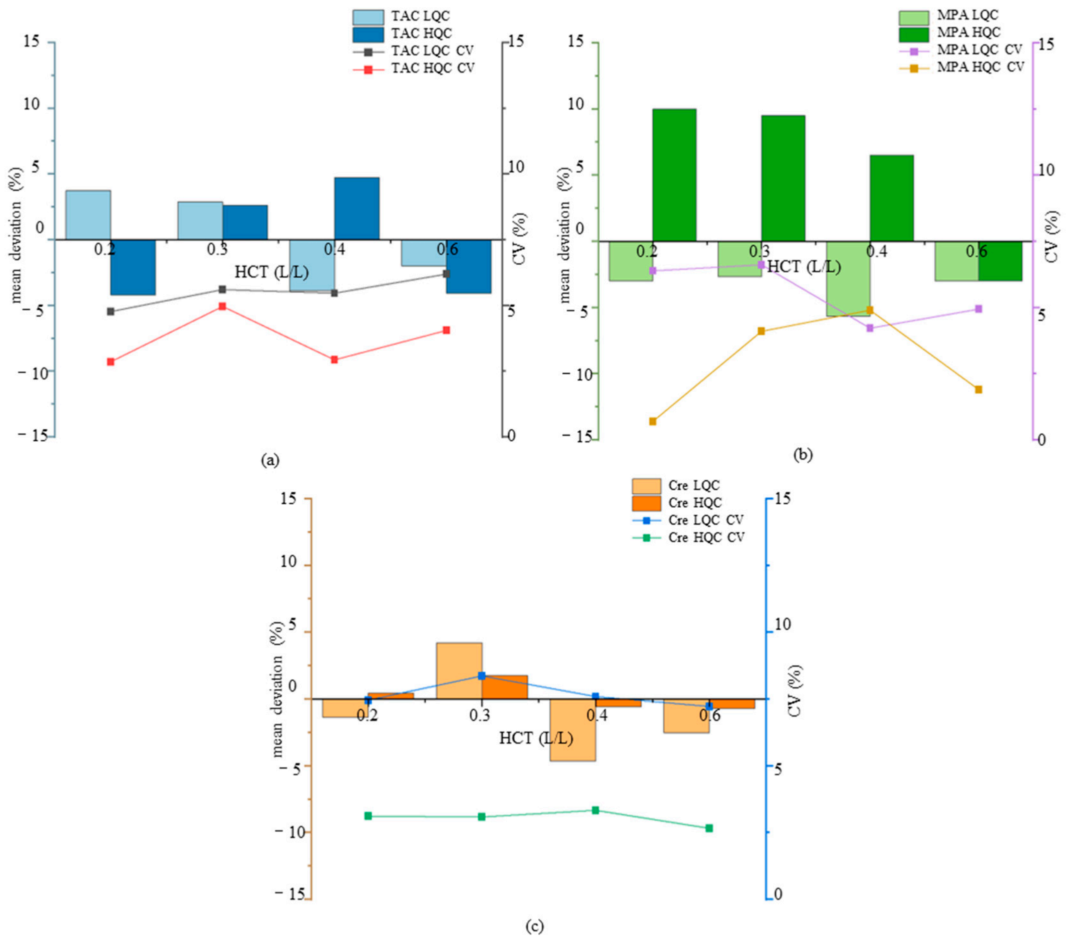

Figure S3. Mean deviation and CV values were obtained from three analytes with different HCT values. (a) TAC; (b) MPA; (c) Cre.

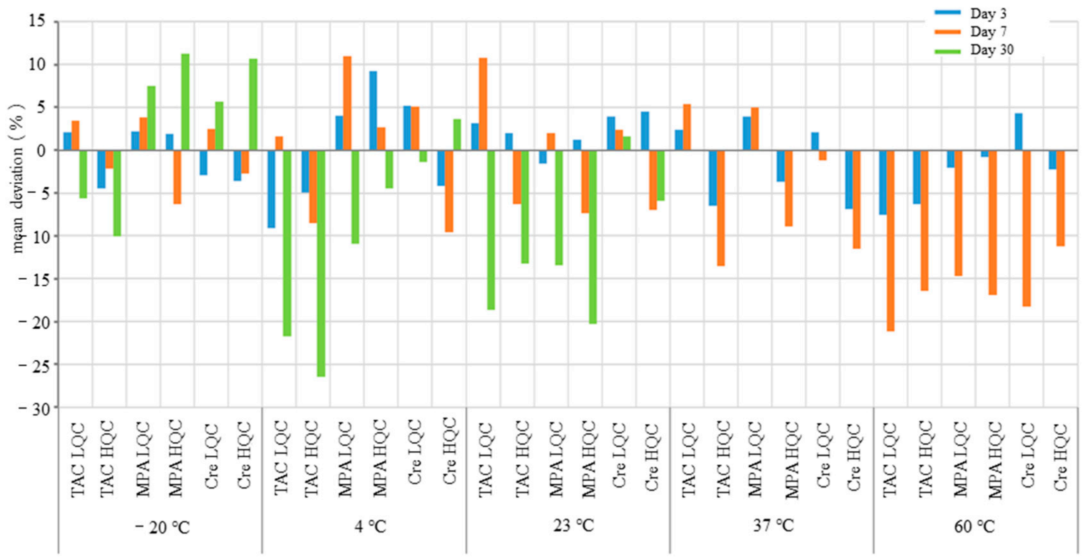

Figure S4. Validation of short- and long-term stability in VAMS samples.
